# Supplementary material for: Are carbon emissions trading and green financial instruments synergistic? -Comprehensive quantitative research based on content analysis
Source: PLoS One. 2024 Mar 7;19(3):e0298601. doi: 10.1371/journal.pone.0298601 (PMC10919601; doi:10.1371/journal.pone.0298601)
Supplement: S1 Data — (ZIP) [file pone.0298601.s001.zip › Data/地级市Z_F工作报告.zip!/║┌┴·╜¡/─╡╡ñ╜¡╩╨/└┤╫╘╣1⁄2╓┌║┼í╛┬φ┐╦╩2╛▌═°í┐_2015.docx]

各位代表：

　　我代表市人民政府，向大会作工作报告，请予审议，并请市政协委员提出意见。

　　一、2014年工作简要回顾

　　过去一年，在省委省政府和市委的正确领导下，在市人大、市政协的监督支持下，我们紧紧依靠全市人民，坚持求实创新、跨越争先，全市经济社会实现持续稳步发展。

　　--尽心竭力稳增长，经济发展稳中有进。积极应对国内经济下行压力，全市地区生产总值实现1300.6亿元、增长7%；固定资产投资实现1048.6亿元、增长17.2%；规上工业增加值实现254.2亿元、增长7.7%；社会消费品零售总额实现475.6亿元、增长13.7%；进出口总额实现120亿美元、下降1.4%（不含绥芬河实现44.1亿美元、增长15.1%）；公共财政预算收入完成88.1亿元、下降3.5%（不含绥芬河完成82.5亿元、增长1.9%）；城镇常住居民人均可支配收入达到24735元、增长13.0%；农民人均纯收入达到16850元、增长13.1%。万元GDP综合能耗下降3.7%，超额完成四种主要污染物减排任务。固定资产投资、社会消费品零售总额、城镇常住居民人均可支配收入增速居全省第一位，地区生产总值、规上工业增加值增速居全省第二位。

　　--积极争取宏观政策，我市在全国全省的战略地位逐步提升。主动对接国家“一带一路”和“五大规划”及国家重大战略部署，我市先后争取到国家老工业区搬迁改造试点、新型城镇化综合试点地区、跨境贸易电子商务服务试点、新能源示范城市试点、全国水生态文明城市建设试点；总投资58亿元的荒沟抽水蓄能电站、总投资12.6亿元的世行贷款高寒城市智能公交系统开工建设，总投资356亿元的哈牡客专在省委省政府直接领导下实现当年立项当年开工，总投资71亿元的哈牡铁路既有线电气化改造项目实现预可研批复，新机场建设获得空军支持启动选址；全市对上争取到位资金突破150亿元。

　　--深入实施产业攻坚，项目建设再上新台阶。全年开复工项目1611个、完成投资786.5亿元，新投产项目达到1214个。大项目投资持续升温，开复工亿元以上项目303个，完成投资增长41.2%，高于全市固定资产投资26.1个百分点。园区功能进一步增强，7个境内园区完成投资142.2亿元、新开工亿元以上项目45个；其中市经开区完成投资69.1亿元、新开工亿元以上产业项目17个；海林经开区获批国家循环化改造示范试点园区。招商引资取得新成效，紧跟省央企民企对接等五大招商活动，新签约亿元以上项目111个、总投资660.7亿元，与中国建材、中国诚通等央企合作项目发展到32个、总投资超过500亿元；全年引进域外资金948.8亿元、增长20.5%。

　　--壮大精品农业，特色现代农业发展步伐加快。粮食生产再创历史新高，总产达到65亿斤。特色农业加快发展，食用菌栽培规模突破40亿袋（块）、棚室蔬菜面积超过5万亩、林下经济产值实现100亿元，分别增长8.1%、8.7%和10%，畜牧业规模养殖比重达到75%以上。农民专业合作社发展到1860个，省级以上龙头企业发展到40户，绿色有机食品认证面积达到450万亩，绿色有机认证产品达到194个。治理水土流失36万亩，造林绿化16.3万亩，田间作业综合机械化率、种植业良种覆盖率分别达到90%和94%。

　　--坚定不移调结构促升级，工业经济稳步发展。工业经济运行总体平稳，工业增加值、主营业务收入增速分别高出全省4.8个和10.2个百分点，非公经济对工业增长贡献率同比提高17.4个百分点，新兴产业增加值增速超过全市工业12个百分点。存量裂变和增量扩张扎实推进，实施亿元以上技改项目34个、完成投资24亿元，恒丰6000吨特种纸等8个项目建成投产。科技对经济贡献作用增强，省级以上企业研发机构达到35家，院士、博士后工作站达到5个，国家级高新技术企业达到19户，石油钻采工具产业基地成为国家级高新技术产业化基地，全市高新技术产业增加值增长13.7%。县域经济发展势头强劲，穆棱、宁安、海林保持全省十强，实现位次前移。

　　--积极推进对外开放，贸旅牵动效应进一步释放。口岸通道更加畅通，中俄海关监管互认机制试运行，开通对俄甩挂运输，华晟国运物流货运量增长1.8倍。对外贸易和进出口加工有新突破，对韩贸易增长56.4%、占全省36.8%，高新技术产品出口增长55%；龙跃经贸合作区、华信农业合作区晋升国家级境外经贸合作区。对外交流合作进一步拓展，与俄滨海边疆区建立定期会晤机制，与我市有经贸往来的国家和地区达到181个。旅游业增势强劲，接待旅游人数突破1700万人次、实现收入108亿元，分别增长20.8%和19%，其中接待境外游客119.3万人次、实现外汇收入5.3亿美元；牡丹江至符拉迪沃斯托克中俄边境12日游线路获国家批准；《智取威虎山3D》、《最后决战》等13部影视作品在我市拍摄，进一步提升了城市影响力。商贸物流持续活跃，万达广场、五洲国际等城市综合体和大型商贸项目开工建设，航空客运量居全省支线机场之首，达到54.3万人次、增长21.7%，其中出入境客运量达到10.6万人次。金融、文化、会展等产业取得新进展，新增地方金融机构8家，金融机构存贷比达到51.2%、同比提高4.8个百分点；渤海靺鞨文化创意产业园被列为国家重点文化产业项目；黑木耳节、远东文博会等展会市场化水平和品牌效应进一步提升。

　　--提升建管理念和标准，城乡面貌发生明显变化。科学编制城市风貌特色、城区水体利用等23个专项规划，牡海公路开工建设，牡丹峰公路、青梅大桥竣工，黄花路西延、宁古塔街等41条道路实现通车，城市框架进一步拉开。认真办理市人大“一号议案”，治理市区交通拥堵的89项任务已完成66项。市区新建改造街头游园13个、新植树木100余万株、新增绿地50万平方米。跨江步行景观桥一期、引牡丹江水进“三溪六湖”等重点工程竣工。实行城市管理“一队式”综合执法改革，各职能部门行政执法职能及部分人员集中到市行政执法局，综合整治占道经营、牌匾广告、私建滥建和“三堆”等现象并取得明显成效。市区清雪全面禁撒融雪剂，清雪机械化率达到71%，清扫保洁标准全面提高。推进城乡统筹发展，构建“1核5城18镇100区”城镇化发展格局，拆迁棚户区284万平方米，改造农危房1.4万户，建成“幸福家园”17个。认真办理市人大“二号议案”，加强市区大气污染治理取得阶段成效，取缔小锅炉264台，淘汰黄标车13094辆，更新环保型公交车200辆；完成节能减排和重点流域治理项目31个，提前完成“十二五”淘汰落后产能任务；污水处理厂二期主体工程完工，城乡生态环境进一步改善。

　　--民生工作扎实有力，和谐幸福牡丹江建设深入推进。全市民生支出达到112亿元，占公共财政预算支出52%，扎实推进28件利民实事，同时又建立了7个“的哥”食堂、提高了环卫工人待遇、成立了法律援助中心、设置了20个公交厕所，“12345”、“12349”热线解决群众生产生活问题18.7万件。就业社保扎实开展，城镇新增就业6.6万人、登记失业率控制在3.4%以内；积极推进社保扩面提标，五项社会保险新增参保6.2万人，企业离退休人员养老金实现“十连增”，市区城乡低保标准分别提高15%和12.5%，新农合参合率达到99.8%。人居环境持续改善，政府出资3.5亿元综合整治老旧住宅小区400万平方米，惠及居民20余万人；出台加强物业管理措施，市区物业管理水平有所提高；廉租房投入使用1515套、7.6万平方米，我市棚改征收经验在全省推广。社会事业全面发展，义务教育标准化学校达到86%，农村学校全部实现集中供热，市一中文科教育成为全省排头，我市学前教育和校园安全工作经验在全省推广；医院标准化建设三年决战圆满收官，实施以“药品零差率销售”为重点的县级公立医院改革试点，医疗远程会诊实现全覆盖；文化惠民和全民健身活动深入开展，评剧《咱们的领袖毛泽东》获第九届中国评剧艺术节参演剧目奖，话剧小品《非诚勿扰》获东北三省戏剧小品大赛导演一等奖和表演金奖；市区开展居家养老服务社区达到94%，西安区被评为全国和谐社区建设示范城区；我市民族团结进步工作获国务院表彰，第三次全国经济普查等28项工作荣膺国家先进。社会整体安全稳定，各类安全生产事故同比下降5.8%；深化公安“十大体系”建设，刑事、治安案件分别下降6.2%和20.9%；在全省率先启动涉法涉诉信访改革试点，信访积案和进京非正常访均下降50%。

　　--深入推进政府改革和依法行政，政府效能进一步提升。深入开展行政审批制度改革，实施企业设立“一表制”，将企业设立所涉及的工商、质监、人民银行、国地税的审批及登记服务集成为“一条龙”，企业行业类许可审批时限精减80%；开通网上行政服务中心，通过互联网受理注册企业315户，全年新增注册企业1826户、增长64.5%，注册资金100万元以上企业885户、同比增长3.8倍，为345户企业免费送照上门；实施建设项目审批“一图制”，一般项目建设、鼓励类重点项目审批时限分别精简33%和50%。哈牡客专在我市涉及22个部门的各种审批只用三天时间全部办结，创造了建设项目审批的“高铁速度”。深化体制、机构和职能改革，取消下放行政审批事项244项，取消行政事业性收费16项，向县（市）重点镇下放事权219项，授予市经开区除规划、土地外的全部市级管理权限；完成市卫生局、计生委机构合并，工商局、质监局职能下划承接和食品药品监督管理体制改革。超前研究法治政府体系建设，在政府法规性文件立法系统化准备、政府依法运行流程建设、政府监督体系建设等方面，创造性开展工作，为法治政府建设奠定了基础。办理人大代表议案3件，建议、批评和意见260件，办理政协委员提案337件，办复率均达100%。持续加强党风廉政建设和作风建设，扎实开展党的群众路线教育实践活动，强化财政资金和政府性投资项目审计监督，“三公”经费支出缩减14.7%。

　　老龄、气象、地震、档案、人防、边防、公积金、民兵预备役、妇女儿童、残疾人、红十字等工作都取得了新成绩。

　　各位代表，过去一年，我们面临的挑战前所未有，取得的成绩来之不易。这些成绩的取得，得益于市委总揽全局、正确领导；得益于市人大、市政协有力监督、鼎力支持；得益于历届市政府打下的坚实基础；得益于全市上下励精图治、不懈奋斗。在此，我代表市人民政府，向全市人民，向人大代表、政协委员，向离退休老领导、老同志，向各民主党派、工商联、无党派人士、人民团体，向驻牡部队和武警官兵，向驻牡中省直单位，向所有关心支持牡丹江发展的各界人士，表示衷心的感谢和崇高的敬意！

　　我们清醒地看到，经济社会发展中还存在不容忽视的问题：经济总量不大、产业结构不优、外贸拉动不强、项目支撑不足等问题仍很突出；城乡建设和管理、城市功能、民生保障、公共服务水平，与百姓期盼还有较大差距；安全生产、公共卫生、环境保护等方面工作还需进一步加强；政府部门顺应和引领新常态发展的能力还需进一步提高；个别干部慢作为、不作为、乱作为的现象仍然存在；经济发展环境不优的问题仍比较突出。对此，我们要高度重视，采取有力措施认真加以解决。

　　二、2015年工作总体部署

　　2015年是全面深化改革、加快法治政府建设的关键之年，是全面完成“十二五”规划的收官之年。在全国经济发展新常态的大背景下，我市正处于产业激烈竞争的挤压期，产业结构调整的阵痛期，深挖潜能释放存量、资源、能源优势的突破期，我市发展既面临新挑战，也有机遇和潜力。

　　从挑战看：一是受国际环境影响，卢布动荡直接影响对俄贸易，长期看汇率波动将影响进出口加工业及相关产业投资；二是国内经济下行压力加大，一般加工业和一般房地产投资意愿将进一步减弱，招商引资竞争将更加依靠产业环境和区位；三是受总需求制约，烟草行业规模、产品结构问题对我市影响很大，一时难以恢复；四是我市产业整体规模不大，龙头企业不强，行业整体竞争优势不突出，在激烈的产业竞争中抗风险能力不强，首控石油等58户规上企业停产，15户骨干企业生产下滑，导致我市工业增速放缓，并短期难以弥补。

　　从机遇和潜力看：一是我市在东北亚的特殊区位及长期对俄经贸、对外交流所形成的经济、文化、合作优势，可以形成新的产业机会；二是国家“一带一路”及“五大规划”发展战略，国务院支持东北地区振兴35条意见和省政府稳增长65条政策措施，为我市在生产力布局、产业结构调整、基础设施改善、解决历史遗留问题、扩大对外开放、发展战略新兴产业等方面提供了重大机遇；三是多年来深化工业立市、金融强市、贸旅牵动战略形成的产业氛围，践行“三实两创”共同价值理念营造的政治生态，为我市发展提供了产业基础和队伍保障；四是我市独特的区位、文化、生态条件，优越的森林、山水、冰雪资源，为旅游、健康、养老服务业发展提供了巨大产业空间；五是去年绥芬河跨境电子商务交易包裹650多万件、增长3.4倍，发展电商及相关产业面临难得机遇。

　　我们要深刻认识经济发展新常态的“四个趋势性变化”，准确把握市委确定的“七个更加注重”的努力方向，满怀激情地抢抓机遇、应对挑战，顺势而为、乘势而上，打造牡丹江经济社会发展的升级版。

　　今年政府工作的总体要求是：深入贯彻党的十八届三中、四中全会精神，贯彻落实习近平总书记系列重要讲话精神，按照省委省政府和市委的决策部署，适应新常态、把握新机遇、争创新优势，坚持稳中求进工作总基调，坚持全面深化改革主线，坚持产业项目建设重点，坚持投资拉动和创新驱动并重，统筹推进经济、文化、社会、生态建设和政府改革，努力在求实创新、跨越争先中彰显更大作为。

　　经济社会发展主要预期目标是：地区生产总值增长6.5%以上，固定资产投资增长10%，公共财政预算收入（可比口径）增长5%，规上工业增加值增长8%，社会消费品零售总额增长12.5%，进出口总额增长3%，城乡居民收入分别增长10%，万元GDP综合能耗下降3.7%，完成四种主要污染物减排任务。

　　三、2015年经济社会发展主要任务

　　新常态下推进经济社会发展，必须立足当前、着眼长远，强化“稳”的措施、明确“转”的方向、突出“改”的主线、加大“惠”的力度，更加有效地稳增长、调结构、促改革、惠民生，推动牡丹江经济社会发展行稳致远。

　　（一）狠抓产业项目建设，做大做强工业经济。紧扣全省“十大重点产业”，深入开展“产业项目提升年”活动，持续增强产业支撑和投资拉动。一是加大项目建设力度。深化新一轮产业项目攻坚，年内3000万元以上项目新开工800个以上、新竣工400个以上，产业项目投资占固定资产投资比重达到50%以上。二是提升招商引资水平。瞄准食品医药生物、高端装备制造、对俄合作、现代服务业等重点产业，突出世界500强、国内行业50强和央企民企等重点目标，广泛开展战略招商、产业链招商、以商招商、以需招商，力争引进亿元以上项目115个，引进域外资金增长15%。三是提升园区载体功能。发挥国家级和省级开发区带动作用，落实飞地政策，打破利益格局，推动园区专业化、集约化、功能化发展。在基础设施“八通一平”的基础上，更加注重强化产业特色、产业链整合、服务功能建设。统筹推进大庆路老工业区搬迁改造试点，加快四城区产业园建设步伐。

　　坚持“工业立市”战略，用足用好国家老工业基地调整改造政策，不断提升工业规模和层次。在存量上，围绕优势产业和骨干企业，推进装备制造产业转型升级、医药行业兼并重组上市、食品企业放大规模、林木林纸产品提升档次，重点促进友搏药业冻干粉、富通空调二期等项目投产达产，加快恒丰纸业、金缘钩缓等骨干企业技术改造。在增量上，工业向园区集中，市经开区开工亿元以上项目50个以上，各县（市）园区开工亿元以上项目50个以上；鼓励全民创业和壮大民营经济，实施中小微企业成长工程，全市规上工业企业发展到500户以上。在结构调整上，以整车、整机、整台套为方向，发展壮大高端装备制造业；强化创新驱动，落实全省千户科技型企业三年行动计划，加强高层次人才引进，支持企业自主创新和产学研合作，鼓励规上企业调整结构向高新企业转型，加快高新技术成果产业化，高新技术产业增加值增长15%以上；加快推进中国诚通生物质能源示范基地、佰佳信和太敬机器人等重点项目，推进北方工具与哈工大机器人集团、新松机器人自动化公司战略合作，培育食品医药生物、智能装备制造两大产业集群。设立生物、机器人等产业发展基金，加大政策扶持力度。

　　（二）大力发展特色精品农业，加快转变农业发展方式。借力国家“两大平原”现代农业综合配套改革，进一步加快发展现代农业。一是壮大新型经营主体，培育新型市场化服务主体，家庭农场、农民专业合作社分别达到1350个、1900个。二是以提质增效增收为主线，做精做优棚室菌、设施菜、高端畜牧业、林下经济等特色优势产业，大力发展外向型农业，全市食用菌栽培规模达到41亿袋（块），蔬菜面积达到40万亩，畜牧业规模养殖比重达到78%以上，林下经济产值达到110亿元，农产品出口基地发展到36万亩。三是加快农业产业化步伐，培育发展龙头企业，提高精深加工比重，推进农业向全产业链扩张，省级以上龙头企业达到46户，加工能力达到180万吨；加快品牌整合，驰名著名品牌、国家地理标志产品分别达到30个和15个。四是加强水利化、农机化、科技化建设，新增和改善灌溉面积35万亩，田间作业综合机械化率达到93%，先进适用技术覆盖率达到91%。

　　（三）加快发展现代服务业，培育壮大新的经济增长点。坚持“贸旅牵动”战略和“金融强市”战略，增强现代服务业对经济增长的拉动作用。一是加快旅游业发展。以镜泊湖、雪乡为龙头，突出湖、林、雪、边、红、俗、特和火山、温泉、湿地、影视等特色旅游，加强经典旅游产品、线路和特色旅游商品开发，提升配套服务整体水平。探索现代营销手段，注重电视、微信、APP应用营销方式，积极开展对俄韩日及港澳台营销。全年接待旅游人数和收入均增长10%以上。二是提升商贸业。突出对俄对韩特色，加快建设万达广场、五洲国际、绿地CBD等大型商业综合体，俄罗斯老街、朝鲜民俗风情街等特色商业街区，亿丰国际汽贸城、牡达农副产品批发等专业市场，推进电子商务与传统商贸融合发展。三是加快发展现代物流业。推进市经开区国际物流产业园、阳明区冷链物流园区等项目建设，重点发展电子商务物流、跨境物流、生产物流、商贸物流，优化生活物流布局。四是大力发展金融服务业和信息服务业。强化金融机构引进，鼓励发展互联网金融、跨境支付等新型金融主体，推进天合石油、友搏药业、富通空调上市进程，完成恒远药业“新三板”挂牌上市和宁安亚麻中小企业私募债发行工作，新增金融机构5家以上。加快发展电子商务，特别是对俄跨境电子商务，打造国家级电子商务示范基地。注重发展新产业业态、新商业模式、新产业平台。

　　（四）加速推进对俄合作转型升级，构筑沿边开放新高地。紧跟国家“一带一路”、龙江及蒙东沿边开发开放带战略，打造龙江陆海丝绸之路“动车组”，加速中俄地区友好合作示范城市建设，与绥芬河联手培育对俄合作新优势。一是做大做实进出口加工。落实省委“出口抓加工、进口抓落地”部署，重点发展绿色食品、消费电子、建材、机电、轻工等出口加工产业，扩大粮、矿、木、油、渔等俄罗斯资源能源进口深加工，加大对俄农业合作，推进越邦果汁生产、圣众俄罗斯海产品加工等一批涉俄项目建设，打造中俄进出口加工产业集群。积极引进对俄进出口百强企业，提升对俄贸易层次。二是不断提升开放功能。以加强口岸通道建设为重点，加快通道向枢纽转变。提升源丰对俄果蔬国际物流、穆棱铁海联运等内陆港功能，推进华晟国运物流中心内陆港及保税物流中心建设，拓展对俄甩挂运输业务。启动牡丹江综合保税区前期工作。三是拓展对外交流合作。深化中俄文化、教育、卫生、体育等领域合作。争取机场落地签或72小时免签政策，推动自驾游、异地办证等跨境旅游业务，提升人文交流的便利性。同时，扩大与韩日、港澳台等国家和地区的交流合作，提升全方位对外开放水平。

　　（五）提升城市功能品位，增强中心城市吸引力和竞争力。坚持“人民城市人民建、人民城市人民管”，让人民群众参与城市建设和管理，全面提高城市规划、建设和管理水平。一是高水平规划城市。抓好新一轮城市总体规划修改，进一步完善综合交通体系、城市道路及景观设计等专项规划编制工作，启动北部、西部、江南新区、沿江等部分区域城市设计，做好城市绿道系统专项规划等20个重点项目规划。二是高质量建设城市。深化城市建设投资方式改革，引入PPP、BOT等投融资模式，多渠道筹措建设资金。加速打造北部、西部、江南新区等重点商圈，加快“三供三治”等公共服务项目建设，进一步完善城市功能。统筹推进高铁新站综合体建设和虹云桥改扩建、太平路南北贯通隧道、西十一条路等跨铁项目，启动建设牡丹江大桥，推进环江路和环城路项目，加快世行贷款高寒城市智能公交系统建设，推进新安街西延与牡海城际路贯通，进一步拓展城市发展空间。加强绿化、亮化、美化，全方位升级城区主街路、广场和桥梁等节点品质，打造城市出入口、主干路与公园立体景观，市区新增绿地50万平方米，启动凤凰山公园建设，进一步提升城市品位。三是高标准管理城市。开展整治市容市貌、占道经营、违法建设、环境污染等10个专项行动，继续推进市区交通拥堵治理，推进智慧城管建设，坚持绿色清雪，让城市面貌有更明显变化。四是结合全国水生态文明城市建设试点，加强生态文明建设。加快牡丹江、穆棱河、绥芬河流域治理，加速推进林海供水工程、奋斗水库建设，完成人工造林2.5万亩、封山育林11.5万亩。打好防治大气污染攻坚战，开展市区燃煤锅炉综合整治，提升清洁能源使用比重。狠抓节能减排和综合治理，推进二发电、热电厂减排项目，完成污泥无害化深度处理项目，生活垃圾处理二期工程投入使用。全面完成“十二五”减排任务。

　　（六）抓住国家级试点机遇，加快新型城镇化建设。做好国家新型城镇化试点，深化体制机制改革。一是高质量编制全市新型城镇化规划，加快城镇基础设施建设，年内城关镇、重点镇完成棚户区改造100万平方米、完成基础建设投资9.6亿元，提高城镇综合承载力；推动产城融合发展，加快响水“两化”等重点项目建设，深化与森工、农垦合作共建，因地制宜发展精品农业、工业加工、商贸物流、口岸服务、旅游文化等特色产业，提升城镇产业支撑能力和吸纳就业能力。二是破解新型城镇化体制机制制约，推进人口管理制度改革；加快农村产权制度改革，全面启动土地承包经营权确权登记和农村宅基地登记颁证工作，推进市、县（市）、乡（镇）三级产权交易联动平台建设；全市城镇化率提高1个百分点以上，农村宅基地使用权发证完成80%，土地流转面积达到30%以上。三是加速推进美丽乡村建设，突出湖光山色、林海人家、雪韵风情、花果长廊、鱼米之乡、民俗风情、红色经典等特色品牌，打造一批生态特色鲜明、产业支撑有力、人居环境优美、乡土气息浓郁的美丽乡村；加强农村道路和饮水安全工程建设，新建改造农村公路300公里，新增农村饮水安全村屯100个、惠及6.7万人。

　　（七）推动文化繁荣发展，持续提升城市软实力。强化文化引领作用，全面加强社会主义核心价值体系建设，弘扬“三实两创”共同价值理念，繁荣地域优秀文化，筑牢牡丹江人文根基。一是发展壮大文化产业，做大做强新闻传媒集团、和音乐器、渤海民族工艺等重点企业，加快建设老工业区文化创意产业园、侨兴新媒体、中俄（牡丹江）文化艺术品交易中心等产业项目，发展壮大广播电视、演艺娱乐、工艺美术、影视服务等重点产业，培育具有地域特征的新型文化业态；加强文化遗产保护与开发，推进数字虚拟渤海国等项目建设。二是大力发展文化事业，深入开展群众性品牌文化活动、送文化下基层活动，继续办好朝鲜族流头节、中俄旅游文化节等活动；鼓励文化精品创作，传播牡丹江正能量和好声音。三是广泛开展全民健身运动，大力发展冰雪运动等优势竞技项目，促进公共体育设施和学校体育场馆面向社会开放。

　　（八）加大保障改善民生力度，保持社会和谐稳定。在经济下行压力下，更加注重民生优先，不断加大民生投入，办好20件利民实事，让百姓更有幸福感、归属感和安全感。一是扎实抓好就业增收工作。落实高校毕业生就业、重点人群援助和创业扶持政策，强化就业服务和技能培训，全年新增城镇就业6万人，城镇登记失业率控制在4%以内。完善企业职工、机关事业单位人员工资正常调整和增长机制，引导农民增加经营收入、劳务收入和财产性收入。二是提高社会保障水平。五项社会保险参保率稳定在90%以上。企业退休人员养老金、城乡居民基础养老金标准分别提高10%和20%。开展城镇居民大病补充医疗保险制度试点。建立低保信息长期公示制度，建立“救急难”和疾病应急救助制度，加强扶贫开发工作。推进居家养老服务工作，实现城市社区开展居家养老服务全覆盖。三是推进保障房建设。积极争取国家棚户区改造扶持资金，推进市区1.1万户棚户区改造，完成回迁安置1万户以上。完成100万平方米老旧住宅小区综合整治。四是加快社会事业发展。鼓励民办学前教育，稳步推进义务教育均衡发展，注重提升市一中文科品牌，提升职业教育与产业发展的融合度，支持驻牡高校发展。深化医药卫生体制改革，稳步推进县级公立医院全面改革，加强新时期爱国卫生工作。五是注重社会治理和管理体系建设。做好村委会换届选举工作。加强街道、乡（镇）等基层组织能力建设，加强社区、村委会等自治组织建设，充分发挥社会自治组织来自百姓、服务百姓的作用，将社会治理与社会服务结合、具体帮助与文化建设结合，构筑为人民服务“最后一公里”的情感供给线和贴身服务链，构建为百姓提供公共服务的无缝组织网络。六是狠抓安全生产工作。加强源头管控，深入开展煤矿、非煤矿山、危险化学品、易燃易爆、道路交通、建筑施工、人员密集场所等重点行业领域安全生产专项整治，严厉查处违规违法行为，严格追究执法监管中失职渎职行为，坚决防范遏制重特大事故。深入开展食品安全源头治理和专项整治。七是全力保持社会稳定。建立信访疑难案件办理机制、重大信访会议调处研判机制和领导包案机制，及时解决群众反映强烈的热点难点问题，全市信访积案下降30%。完善突发事件监测预警体系，提高预防和处置能力。八是深化平安牡丹江建设。推进公安基础信息化、警务实战化、执法规范化、队伍正规化建设，深入开展“三大行动”，加强警务巡防巡控，严厉打击和严密防范各类违法犯罪活动。

　　（九）科学编制“十三五”规划，统筹当期建设和长远发展。立足实现全面建成小康社会目标，深度对接国家“一带一路”及“五大规划”发展战略，系统总结“十二五”规划执行情况，完成“十三五”规划编制工作。积极对接国家及省“十三五”规划编制工作，在扩大对外开放、门户城市建设、对俄功能区和经济带、各种改革及政策试点、生态文明建设、重大基础设施建设、战略新兴产业发展、解决历史遗留问题等方面，有更多项目工程纳入国家和省“十三五”规划。

　　突出重大项目工程谋划推进，力争东乌铁路、牡符高速铁路、牡佳快速铁路、牡宁城际高速路、机场迁建、对俄管线对接、利用俄远东港口等重大项目取得实质进展，促进哈牡客专、哈牡铁路既有线电气化改造、荒沟抽水蓄能电站、牡绥铁路扩能改造等重点项目加快建设。

　　四、全面加强政府自身建设

　　全面贯彻党的十八届四中全会精神和省委省政府、市委战略部署，加强科学民主法治的服务型政府建设，提升政府执行力和公信力，为牡丹江跨越争先发展提供坚实保障。

　　加快法治政府建设。落实全面推进依法治国战略，完善政府依法行政体系，健全决策、管理、监督机制。配合市人大做好重点领域的立法工作，建立市政府可系统遵循的法规体系。健全依法决策机制，把公众参与、专家论证、风险评估、合法性审查、集体讨论决定确定为重大行政决策法定程序，建立重大决策终身责任追究制度和责任倒查机制，建立各级政府法律顾问制度、专家咨询论证制度。深化行政执法体制改革，重点在城市管理、市场监管、资源和生态保护等领域推行综合执法。完善行政执法和司法衔接机制。强化对行政权力的制约和监督，打造行政大监督平台，将党内监督、人大监督、民主监督、行政监督、审计监督、司法监督、社会监督、舆论监督系统化、立体化、法治化，完善纠错问责机制，防止权力滥用和诿责。落实“谁执法、谁普法”责任制，推进政府系统学法用法常态化，全面提高法治观念和治理能力。

　　加强服务型政府改革。深入推进重点领域改革，打造精简统一高效、集中集约集成的服务型政府。集成市政府审批职能，入门审批按分钟、建设审批按小时计算时间，加快审批速度，实行牵头部门联审会办、一章办结。集中中介机构服务，集成服务内容，降低服务资费。集约政府职能，按照一件事一个部门的原则，归并部门职能，解决政出多门、职能交叉问题；加快电子政务建设，用信息化进行政府流程再造，深度集成政府职能；整合土地、房产、林地、草原等产权管理职能，实施不动产统一登记制度改革。统筹推进财税体制改革，启动预算管理制度改革，加强地方政府债务管理，推进公共资源配置市场化改革，拓展政府购买服务领域和事项。

　　着力优化发展环境。建立和规范“三个清单”，依法公开政府“权力清单”和“责任清单”，切实做到法无授权不可为、法定职责必须为。建立市场“负面清单”，实现市场准入的公平一致，做到清单之外无审批、法无禁止即可为。维护企业经营秩序，建立门禁制度，除安全监管、环境保护和食品药品监督等事项外，其他入企检查收费行为实行向市级主管领导报备登记制。充分发挥企业投诉中心作用，对企业投诉的慢作为、不作为、乱作为等问题一律严格问责。全面落实涉企收费减免政策，加强涉企部门收费监管。推进政府信用管理，对特许经营、购买服务、政府采购等事项实行合同化和备案制管理；对企业和投资者，突破政策规定的不承诺，超越法律法规的不承诺，以政府诚信带动社会诚信建设。

　　加强廉政建设。落实全面从严治党的要求，坚持“三严三实”，全面提升政府执行力。弘扬求实、务实、落实的实干精神；弘扬敢试敢闯、勇于改革、创新创一流的争先精神；弘扬做事依法、做人守法的法治精神；弘扬专业、专注、专心的职业精神；强化事不隔夜、事必躬亲、事无巨细的责任意识，时刻保持良好的工作状态。巩固党的群众路线教育实践活动成果，坚持不懈地反对“四风”，以零容忍态度遏制腐败。严控会议规模和文件数量，严控“三公”经费；严格落实“一岗双责”，健全完善反腐倡廉制度体系，严惩各种违法违纪行为，以清正廉洁的形象赢得人民群众的支持和信任。

　　各位代表，全市人民对牡丹江加快发展寄予厚望，经济发展新常态迫切需要彰显新作为。让我们在市委的正确领导下，在市人大、市政协的监督支持下，坚定信心、开拓进取，勇于担当、攻坚克难，奋力开创牡丹江持续健康发展新局面！
